# Current and Emerging Therapeutic Strategies for the Treatment of Duchenne Muscular Dystrophy

**DOI:** 10.3390/genes17050533

**Published:** 2026-04-30

**Authors:** Miguel A. Lopez Perez, Noah L. Weisleder

**Affiliations:** 1 Department of Physiology and Cell Biology, The Ohio State University, Columbus, OH 43210, USA; lopezperez.3@osu.edu; 2 Department of Molecular and Cellular Biochemistry, University of Kentucky College of Medicine, Lexington, KY 40508, USA

**Keywords:** Duchenne muscular dystrophy, viral vectors, non-viral therapy, gene editing, membrane repair, therapeutics

## Abstract

**Background/Objectives**: Duchenne muscular dystrophy (DMD) is a fatal, progressive neuromuscular disorder caused by mutations in the dystrophin gene, leading to the absence of functional dystrophin protein. As the largest gene in the human genome, the *DMD* locus is highly susceptible to mutations, contributing to a prevalence of approximately 1 in 3800–6300 live male births worldwide. This review aims to provide a comprehensive and critical synthesis of current and emerging therapeutic strategies for DMD. **Methods**: We conducted a narrative review of the literature, integrating findings from clinical trials, regulatory approvals, and preclinical studies. We categorized therapeutic approaches into mutation-agnostic and mutation-specific strategies, with emphasis on the mechanism of action, clinical progress, and translational limitations. **Results**: Current standards of care, including corticosteroids and supportive interventions, remain foundational in disease management. Mutation-specific approaches such as exon skipping and adeno-associated virus (AAV)-mediated gene replacement can restore dystrophin expression, although clinical benefit remains variable and is influenced by factors such as mutation type, delivery efficiency, and durability. Emerging genome editing strategies offer the potential for permanent correction but face significant challenges related to delivery, safety, and scalability. Emerging mutation-agnostic therapies targeting inflammation, fibrosis, and membrane instability provide broader applicability but do not directly address the underlying genetic defect. Across modalities, key limitations include modest functional outcomes, safety concerns, and variability in clinical trial endpoints. **Conclusions**: The DMD therapeutic landscape is rapidly evolving, and future progress will likely depend on optimizing delivery platforms, improving durability, and integrating combination strategies to address the multifaceted nature of disease progression.

## 1. Introduction

Duchenne muscular dystrophy (DMD) is a severe, progressive neuromuscular disorder characterized by the gradual deterioration and weakening of skeletal and cardiac muscle tissue [1]. DMD is caused by mutations in the dystrophin (*DMD*) gene, which result in either the complete loss of dystrophin protein expression or the production of a smaller, partially functional dystrophin [2,3]. As an X-linked disorder, DMD primarily affects male patients. Due to the large size of the *DMD* gene (~2.5 Mb), it is highly prone to spontaneous mutations, which contribute to its high prevalence, with an estimated incidence rate of 1 in 3800 to 6300 live male births. This makes DMD the most common lethal monogenic disorder worldwide [4,5,6]. Early clinical manifestations typically appear between ages 3 and 5, often including delayed motor milestones, gait abnormalities, or difficulties with running and climbing [7]. Disease progression is unrelenting and variable; most patients lose ambulation by 10–12 years of age, followed by progressive respiratory insufficiency and cardiomyopathy during adolescence and adulthood [8,9]. Historically, life expectancy rarely exceeded the early twenties; however, current standards of care, particularly corticosteroid use, assisted ventilation, and heart failure management, have extended survival into the third and fourth decades, with some individuals living beyond 40 years of age [10].

DMD was first described in the literature in the mid-1800s, with the first known case reports provided by Giovanni Semmola (1834) and Gaetano Conte (1836), and the most detailed early reports provided by Guillerme-Benjamin-Amand Duchenne (1861) [11]. However, dystrophin would not be identified as the protein product of the *DMD* gene until the late 1980s [12]. The *DMD* gene was discovered as, and continues to be, the largest known gene in the human genome [12]. Spanning 79 exons and producing a 427 kDa cytoskeletal protein, the *DMD* gene is vulnerable to spontaneous mutations that disrupt the open reading frame. Out-of-frame deletions, duplications, or nonsense mutations abolish full-length dystrophin expression and result in the classic Duchenne phenotype, whereas in-frame mutations may generate a lower molecular weight, partially functional protein associated with the milder Becker muscular dystrophy [13]. Functionally, dystrophin interconnects the intracellular cytoskeleton to the extracellular matrix through the dystrophin-associated protein complex at the muscle cell plasma membrane (sarcolemma). This crucial anchoring provides mechanical stability during routine muscle contraction and relaxation [14,15]. The absence of dystrophin renders skeletal and cardiac muscles highly susceptible to contraction-induced injury, ultimately triggering the characteristic inflammation–necrosis cascade of pathophysiology observed in DMD [16].

Loss of dystrophin has several effects on muscle cells, including the disruption of sarcolemma integrity, due to a higher propensity for recurrent membrane tears and cycles of myofiber necrosis [15]. These tears compromise the barrier function of the sarcolemma so damaged fibers release intracellular contents that trigger an inflammatory response, initially dominated by neutrophils and macrophages, which further exacerbate tissue injury by generating reactive oxygen species and proteolytic enzymes [17,18]. Although muscle cells possess a robust plasma membrane repair response, their repair capacity cannot meet the extent of damage sustained in weakened DMD muscles [19]. Chronic inflammation drives the expansion of fibroblasts and adipogenic precursors, leading to the widespread deposition of extracellular matrix components and intramuscular fat [20]. This inflammation–necrosis cycle ultimately leads to the replacement of functional muscle with fibrotic and adipose tissue, a hallmark of DMD disease progression. Due to the severity and extent of skeletal muscle affected, therapeutic intervention has been challenging, particularly for transgene delivery approaches.

The therapeutic landscape for DMD has expanded rapidly over the past three decades. Early disease management relied almost exclusively on supportive care, with corticosteroids emerging in the late 1980s and early 1990s as the first pharmacological intervention shown to prolong ambulation, delay respiratory or cardiac decline, and extend lifespan [21]. Steroid therapy demonstrated significant promise as a first-line therapy; however, it displayed a limit to therapeutic benefit and developed significant adverse side effects with long-term use (weight gain, osteoporosis, cataracts, etc.) [21]. The 2010s ushered in a paradigm shift with the development of genetic medicines, including antisense oligonucleotide (ASO) exon skipping, and micro-dystrophin gene replacement therapy mediated by adeno-associated virus (AAV) vectors [22,23]. Since the inception of genetic modalities, several ASO-based therapies and one AAV gene therapy have gained conditional accelerated regulatory approval [24]. More recently, genome-editing tools such as CRISPR/Cas9 have been estimated to have the potential to correct >60% of deletion mutations associated with DMD, suggesting the possibility of a one-time intervention that could correct the primary genetic mutation [25]. As DMD therapies continue to broaden, such treatment approaches can be grouped into two principal classes: mutation-specific and mutation-agnostic interventions. While one approach centers on correcting a patient’s specific genetic mutation through the use of precision medicine, the other attempts to address more fundamental deficiencies within the disease pathology, such as a fragile sarcolemma.

Given the pace of therapeutic development seen in just the last decade, a comprehensive analysis of current and emerging strategies is warranted. This review will summarize standards of care and first-line pharmacological interventions that remain foundational in DMD patient care (Table 1). We will then examine the growing portfolio of genetic medicines, including those already approved, and specific candidates at various stages of the developmental pipeline. We will discuss genetic tools such as exon skipping, viral and non-viral gene replacement, and genome editing platforms, with an emphasis on mechanisms, clinical progress, and technological challenges. Lastly, we will examine emerging modalities targeting both upstream and downstream pathology, such as membrane protectants, anti-inflammatory approaches, and antifibrotic agents. Collectively, this review aims to contextualize the current therapeutic landscape for DMD, critically evaluate the perceived and observed benefits, including limitations of existing technologies, and offer a perspective across the various therapeutic strategies and the potential future directions of the field.

## 2. Mutation Agnostic Strategies

### 2.1. Supportive Care and Management

While recent innovative genetic tools have shown promise, supportive care remains a cornerstone of DMD management. Because disease progression primarily affects skeletal and cardiac muscle, considerable attention has been given to preserving respiratory and cardiac function throughout the patient’s life. Routine pulmonary function monitoring, lung volume recruitment, manual and mechanically assisted coughing, and non-invasive ventilation have been shown to improve quality of life and prolong patient survival [26]. Recent recommendations have increased the focus on anticipatory respiratory management before complications develop and have highlighted the benefit of standardizing respiratory care to accurately evaluate treatment effects in clinical trials [27]. The standardization of DMD respiratory profiles has been proposed to address potential flaws in the existing studies that rely on overly pessimistic historical controls to generate statistically significant results [28,29]. Taken together, current and historical treatment guidelines underscore respiratory management as a fundamental component of care for patients with DMD.

As respiratory management has become routine, cardiac complications now account for an increasing proportion of life-limiting outcomes in DMD. Cardiomyopathy is a nearly universal clinical feature of DMD and requires proactive monitoring and management. Cardioprotective medications (e.g., ACE inhibitors, beta-blockers) and device implantation are at times necessary [30,31]. While these supportive measures do not address the underlying genetic defect, they are crucial for delaying degeneration and thereby prolonging the patient’s life and health span.

Preserving mobility has also been shown to delay disease progression and reduce the risk of secondary complications. Physical therapy, as well as other supportive allied modalities, plays a critical role in extending muscle function and improving quality of life. Regular stretching, range-of-motion (ROM) exercises, orthotic support, and orthopedic management help prevent contractures, maintain function, and reduce the impact of scoliosis on respiratory capacity [26,32]. Representative examples of available supportive care modalities are summarized in Table 2.

Several updates to standards of care have supplemented the 2018 care considerations for DMD, highlighting the increasingly complex therapeutic landscape in contemporary DMD care. These updates underscore the importance of multidisciplinary rehabilitation and provide improved guidance on the timing, frequency, and integration of physiotherapy with pharmacological and genetic interventions [33]. As novel therapeutic strategies evolve, supportive devices and rehabilitation remain an indispensable component of comprehensive DMD care. Together with emerging therapies, they will be critical to maximizing patients’ functional and survival outcomes.

### 2.2. Corticosteroids

Corticosteroids remain the first-line pharmacological intervention for DMD [32]. As a mutation-agnostic approach, long-term steroid therapy has benefited a vast majority of the DMD patient population, independent of their specific genetic mutation [32]. With decades of clinical evidence supporting their use, prednisone and deflazacort have been widely utilized to slow disease progression, preserve muscle strength, maintain ambulation, retain pulmonary function, delay the onset of cardiomyopathy, and, most importantly, increase survival [21,26,34,35]. Although both prednisone and deflazacort have consistently been shown to improve aspects of DMD clinical manifestations to a similar degree, each exhibits a unique side effect profile. Notably, prednisone acts on both the glucocorticoid receptor (GR) and the mineralocorticoid receptor (MR), whereas deflazacort exhibits more selective glucocorticoid receptor activity. This dual receptor activation by prednisone has been associated with an increased risk of certain adverse effects, including fluid retention, hypertension, and metabolic disturbances, which may limit tolerability in some patients [35]. While weight gain and behavioral side effects appear to be worse with prednisone, growth parameters and cataracts appear to be of greater risk with deflazacort [35,36]. Several dosing regimens have been studied, with differences based on regional practices, patient age, and comorbidities [37,38]. Still, the doses most commonly used, based on risk–benefit evaluations, are ~0.75 mg/kg/day for prednisone or 0.9 mg/kg/day for deflazacort [36,39].

Long-term steroid therapy does not come without cost. Chronic use of corticosteroids is often associated with adverse events, including weight gain, the inhibition of linear growth, behavioral issues, puberty delay, bone density loss, cataracts, metabolic disturbances, and an increased susceptibility to vertebral fractures [40]. Of particular concern is the suppression of the hypothalamic–pituitary–adrenal (HPA) axis, which can lead to adrenal insufficiency and inhibit endogenous cortisol production [32,41]. The management of steroid side effects is critical, as abrupt reduction or withdrawal of long-term corticosteroids can result in adrenal crisis [32]. Despite these adverse effects, the real-world functional improvements and survival benefits have established corticosteroid therapy as a cornerstone of DMD care. It will continue to be utilized in conjunction with more novel therapeutic interventions.

### 2.3. Dissociative Steroid Therapy

Recently, vamorolone (VBP15) has been introduced as a promising next-generation corticosteroid, approved for use by the U.S. Food and Drug Administration (FDA) under the brand name AGAMREE. It is reported to have efficacy similar to that of current corticosteroids used in the treatment of DMD, with an improved side-effect profile [42]. Vamorolone is a dissociative steroidal anti-inflammatory drug, designed to retain anti-inflammatory and muscle-stabilizing benefits while reducing the negative side-effects commonly associated with long-term steroid therapy, particularly on growth, bone health, and metabolic regulation [43].

Much of the guidance for the use of vamorolone was taken from the pivotal VISION-DMD clinical trial, a 24-week phase 2b, randomized, double-blinded, placebo- and prednisone-controlled trial that included a 24-week treatment extension period [44]. In this trial, vamorolone and prednisone were compared in 121 boys with DMD aged 4 to 7 years. After 24 weeks, both treatments showed improvement across all muscle-function outcomes. However, only vamorolone showed no incidences of weight gain or behavioral, immune-related, gastrointestinal, or skin or hair changes. This trial demonstrated that the main benefits of vamorolone were likely reduced bone morbidities and behavioral issues. Moreover, after the 24-week extension period, the efficacy of vamorolone was maintained at 48 weeks while displaying a more favorable safety profile [45]. Early observational data suggest that vamorolone may present a functional alternative to classical corticosteroids, potentially offering DMD patients a long-term steroid therapy option with fewer skeletal and endocrine complications [46].

Following its approval in 2023, vamorolone adoption continues to expand for the treatment of DMD in patients aged 2 years and older [47]. Its introduction underscores a broader shift toward patient-centered therapies that aim to decouple the anti-inflammatory efficacy of corticosteroids from their characteristic systemic toxicities. By selectively modulating glucocorticoid receptor activity, vamorolone serves as a more tolerable alternative, particularly for patients where growth suppression, compromised bone health, and behavioral side effects are primary clinical considerations.

However, the evidence base for vamorolone remains less mature than the decades of data supporting traditional glucocorticoids. While initial results are promising, its long-term comparative effectiveness and impact on cardiopulmonary outcomes remain to be fully defined. Continued longitudinal studies will be essential to determine the optimal positioning of vamorolone relative to prednisone and deflazacort as the standard of care evolves across different stages of disease progression. Representative examples of contemporary steroid therapy utilization are summarized in Table 2.

## 3. Mutation-Specific Strategies

While current first-line treatment options primarily aim to mitigate the secondary pathophysiological symptoms of DMD, recent novel therapies have been developed that directly target the genetic basis of the disease, namely the lack of dystrophin production.

### 3.1. Exon Skipping

Exon skipping is one of the most clinically advanced genetic therapies for the treatment of DMD. This approach utilizes antisense oligonucleotides (ASOs) designed to bind pre-mRNA transcripts and modulate splicing to skip specific exons. In DMD, excluded exons contain out-of-frame mutations, which when avoided, restore the translation reading frame of the dystrophin gene [48]. By converting an out-of-frame mutation into an in-frame transcript, exon skipping allows for the production of a shortened but still partially functional dystrophin protein, comparable to that observed in Becker muscular dystrophy. Although dystrophin expression can be achieved through exon skipping, it does not alter the genome and therefore requires repeated administration to maintain efficacy.

Four exon skipping therapies have received accelerated approval from the U.S. FDA. Eteplirsen (Exondys 51), which induces the skipping of exon 51, was the first approved in 2016 and is applicable to approximately 13% of the DMD patient population [23,49]. Subsequent approvals expanded the therapeutic scope to additional mutations, including Golodirsen (Vyondys 53) in 2019 and Vitolarsen (Viltepso) in 2020, both targeting exon 53 and estimated to address ~8% of patients each [50,51]. More recently, Casimersen (Amondys 45) was approved in 2021 for exon 45 skipping, addressing another ~8% of individuals with DMD [52]. Together, these therapies marked a major milestone in the genetic manipulation of DMD, demonstrating that splice modulation could be translated from a molecular concept to clinical application.

Despite receiving accelerated approval, several challenges limit the overall impact of this clinically established antisense oligonucleotide (ASO) platform. Although originally conceptualized in the late 1970s, its application in DMD has only recently matured into clinically validated treatments [53]. One major hurdle is the relatively low level of dystrophin expression that is observed in treated patients. Quantitative analyses of muscle biopsies repeatedly report dystrophin levels of only 1–5% of healthy dystrophin expression, raising questions about the degree of long-term functional benefit that can be expected [54]. The high degree of variation in dystrophin expression across muscle fibers in the same muscle group, contralateral muscle groups, and within cohorts has also made it challenging to construct an accurate risk–benefit profile from human clinical trials [55]. Additionally, exon skipping is a mutation-specific tool and therefore each ASO product is limited to only the subset of DMD patients that harbor the targeted mutation. This restriction leaves a substantial proportion (>70%) of DMD patients ineligible for currently approved exon skipping therapies, limiting the overall reach of this approach [48].

The practical functional outcomes associated with exon skipping remain a subject of ongoing debate. Although consistent increases in dystrophin expression are observed, the link between this biochemical improvement and significant gains in motor function, ambulation, or cardiopulmonary health has been modest and variable across studies [56,57,58]. Furthermore, the logistical and economic challenges of lifelong, repeated intravenous treatments limit the practical utility of current ASO products.

Ultimately, exon skipping represents a validated yet inherently limited strategy within the DMD treatment landscape. Its current role, defined by a robust biomarker signal that does not always scale to clinical benefit, positions it as an effective yet incomplete solution. Despite these limitations, the platform has established a clinically relevant proof- of-concept for molecularly targeted therapy, providing a foundational framework that continues to guide the development of next-generation splicing modulators with optimized potency, tissue penetration, and durability [59,60]. The ASO therapies discussed in this section are summarized in Table 3.

### 3.2. Adeno-Associated Virus (AAV)-Mediated Gene Replacement

As a seemingly straightforward approach, gene replacement therapy aims to deliver a functional dystrophin gene to affected skeletal and cardiac muscles to restore sarcolemma integrity and decrease mortality. Adeno-associated viral (AAV) vector delivery systems have become the platform of choice in DMD [61]. However, due to the large size of the *DMD* coding sequence (~14 kb), full-length dystrophin cannot be packaged into conventional AAV vectors. Researchers have therefore developed truncated “micro-dystrophin” and “nano-dystrophin” constructs that retain key functional domains and can be accommodated within AAV capsids [62]. AAV vectors are non-integrating, muscle-tropic delivery systems that have historically been viewed as having a comparatively favorable translational profile for in vivo muscle gene delivery, thus they have become the platform of choice for systemic gene delivery in DMD [63]. Gene replacement therapies discussed in this section are summarized in Table 3.

#### 3.2.1. The Only FDA-Approved Gene Therapy for Duchenne Muscular Dystrophy

In June 2023, the FDA granted Sarepta Therapeutics accelerated approval for delandistrogene moxeparvovec (brand name Elevidys) for the treatment of DMD in ambulatory boys aged 4 to 5 years [24]. It was the first, and remains the only, conditionally approved gene replacement therapy for DMD. This AAVrh74-based therapy delivers a micro-dystrophin transgene systemically and is indicated for patients with confirmed *DMD* mutations amenable to this approach. Approval was based on evidence of micro-dystrophin expression in muscle biopsies and favorable safety signals in early clinical studies [64]. In June 2024, data from the EMBARK trial (NCT05096221) was used to expand the indications to include a broader age range (4 years and older) and both ambulatory and non-ambulatory patients [65]. However, subsequent safety findings reported in 2025 prompted label revisions restricting its use to ambulatory patients aged 4 years and older only, removing the non-ambulatory indication. This revision followed reports of serious liver injury and treatment-associated fatalities in non-ambulatory patients. These developments underscore the evolving safety profile of systemic AAV gene therapies and highlight the importance of continued post-marketing surveillance [66].

#### 3.2.2. Gene Replacement Programs in Development

Several additional AAV micro-dystrophin programs are in various phases of clinical development:

Fordadistrogene Movaparvovec (PF-06939926) from Pfizer: This AAV9-based micro-dystrophin program demonstrated early promise, but Pfizer announced discontinuation of PF-06939926 in July 2024 following safety concerns, including serious adverse events in later-stage trials (Pfizer 2024; company press release). The phase 3 CIFFREO study (NCT04281485) was halted, underscoring the safety challenges of systemic first-generation AVV capsid-mediated gene delivery in DMD. Pfizer also cited failure to achieve the primary endpoint as the reason for discontinuing development. A recurrent theme in gene replacement trials.

RGX-202 from Regenxbio: RGX-202 employs the NAV AAV9 vector platform and is currently being evaluated in the phase 1/2 AFFINITY DUCHENNE trial (NCT05693142), which is actively recruiting. Initial safety and expression data will be critical for advancing this candidate therapy.

SGT-003 from Solid Biosciences: Tested in the INSPIRE DUCHENNE phase 1/2 study (NCT06138639), SGT-003 is another AAV9 micro-dystrophin gene therapy with early interim data reported (Solid Biosciences 2024; company update). The trial remains active and recruiting, with continued monitoring of expression and functional outcomes.

GNT-0004 from Genethon: In Europe, GNT-0004 has advanced into a phase 3 trial (2023-505187-11-00) that is actively recruiting in France. This program’s design emphasizes robust efficacy and functional outcome assessments, contributing to the growing evidence base for gene replacement strategies outside the United States.

Across these programs, the systemic delivery of truncated dystrophin transgenes has consistently led to detectable protein expression in skeletal muscle biopsies and the preservation of components from the dystrophin-associated protein complex, critical for dystrophin’s structural role. While early clinical data suggest trends toward the stabilization of motor function, as measured by the North Star Ambulatory Assessment (NSAA), the 6-minute walk test (6MWT), and other timed assessments, formal statistical significance continues to vary across studies [64]. Importantly, cross-program comparisons are confounded by non-uniform trial designs, varying patient baselines, inconsistent corticosteroid regimens, and divergent endpoint selection. As a result, detectable micro-dystrophin expression has yet to be established as a reliable surrogate for predicting functional benefit across different delivery platforms. While there are efforts by the field to address these challenges [67], comparative assessments between different genetic therapies will benefit from more standardized approaches. Furthermore, longitudinal data correlating this expression with durable improvements in pulmonary and cardiac parameters remain limited and will be critical for defining the full therapeutic impact of AAV-mediated gene replacement.

#### 3.2.3. Safety, Immunogenicity, and Persistence Challenges

Despite promising results, several immunological and physiological challenges have tempered the clinical enthusiasm for AAV-based gene replacement therapies. Safety concerns extend beyond transient liver or cardiac toxicity to include severe immune-mediated reactions, such as complement-activated thrombotic microangiopathy (TMA) and systemic inflammatory responses [68,69]. Following the expanded use of delandistrogene moxeparvovec (Elevidys), reports of treatment-associated fatalities have prompted intense scrutiny regarding the risks of high-dose systemic administration. These risks are further complicated by pre-existing and treatment-induced circulating neutralizing antibodies, which limit vector redosing. Moreover, the delivered non-integrating episomal transgene is at risk of loss over time due to natural muscle turnover [25,70].

Consequently, the field has entered a more cautious phase in which biological proof of mechanism (detectable dystrophin expression) is no longer sufficient to justify the risk–benefit profile of AAV delivery. While the accelerated approval of Elevidys validated the underlying therapeutic principle of micro-dystrophin expression, the reliance on this surrogate biomarker highlights the gap between biochemical rescue and demonstrable functional improvement [71]. Moving forward, the clinical utility of systemic AAV-delivered gene therapies will be defined by their ability to overcome at least three hurdles: the mitigation of high-dose innate and adaptive immune triggers, the stabilization of functional outcomes across diverse patient cohorts, and the long-term persistence of the transgene in the target tissue [72]. Ultimately, establishing a manageable safety profile that is devoid of acute toxicities observed in recent high-dose trials will be required to transition these programs from conditionally approved/experimental interventions to a sustainable standard of care.

### 3.3. CRISPR/Cas Gene Editing

Gene editing has emerged as a transformative, mutation-specific therapeutic strategy for DMD, with the potential to restore dystrophin expression from the endogenous locus through permanent genome modification [73,74]. In contrast to exon skipping, which requires repeated dosing, gene editing aims to achieve a durable genetic correction that can be sustained over time in terminally differentiated muscle tissue [75]. The most widely explored platform is the CRISPR/Cas9 editing system, which enables targeted DNA cleavage and subsequent repair that can restore the open reading frame (ORF), delete mutation-bearing regions, or (less commonly) precisely correct a specific pathogenic variant.

CRISPR/Cas editing systems use a single guide RNA (sgRNA) to target the Cas nuclease to a complementary genomic sequence adjacent to a protospacer-adjacent motif (PAM). At the recognition site, the nuclease introduces a double-strand DNA break (DSB) that is subsequently repaired by endogenous DNA repair pathways, such as non-homologous end joining (NHEJ) or homology-directed repair (HDR). While gene editing ideally utilizes HDR to integrate a corrected DNA template, the more common yet error-prone cellular repair mechanism activated is NHEJ. HDR is significantly inefficient in target muscle tissue due to limited proliferation. Therefore, most current gene-editing approaches rely on NHEJ to generate reframing insertions and deletions (indels) or to extract defined genomic segments to restore a functional transcript [76]. Early proof-of-concept studies have demonstrated that CRISPR-based excisions of the mutated region in the *mdx* mouse model can restore dystrophin expression and improve muscle physiology following local or systemic delivery, supporting the feasibility of editing skeletal and cardiac muscle in vivo [73,74,75,77].

Various corrective genetic strategies have been pursued to address the heterogeneity of *DMD* mutations. ORF reframing strategies typically use a single cut near a splice site or within an exon to induce indels that restore the downstream reading frame, thereby enabling dystrophin translation from an internally modified locus [73]. Alternatively, exon-deletion approaches use paired sgRNAs flanking one or more exons to remove mutation-bearing segments, producing an in-frame transcript analogous to a partially functional Becker-like dystrophin [78]. This approach leverages the wide versatility of the CRISPR/Cas9 editing platform, which allows for the multiplex correction of mutant exons, with an estimated capacity to correct up to 62% of *DMD* mutations [78]. In parallel, single-nucleotide precision correction has also been explored. However, cellular HDR remains infrequent in postmitotic muscle, prompting the development of gene-editing modalities that aim to circumvent the need for DNA breaks and donor templates.

#### 3.3.1. Base Editors

To eliminate the need for a DNA DSB and reduce the high error rate of NHEJ, more recent base editing platforms have been introduced. This new technology fuses catalytically impaired Cas nucleases to deaminases, enabling programmable single-base conversions without generating breaks in the target DNA sequence [79,80]. The ability to specifically modify single nucleotides is especially relevant to DMD, where many pathogenic variants are nonsense mutations or splice-disrupting changes that are amenable to precision base conversion [81].

Recent DMD preclinical studies have reported the use of adenine base editing to directly correct nonsense mutations in vitro by rescuing dystrophin expression in cardiomyocytes derived from DMD-patient-induced pluripotent stem cells. Additionally, the same study demonstrated in vivo correction in a humanized DMD mouse model using both local and systemic delivery of gene-editing complexes [82]. Other groups have taken a different approach, targeting splice sites to induce exon skipping for therapeutic benefit. Lin et al. restored robust dystrophin expression in cardiac and skeletal muscle by systemic delivery of base editors via AAV in a humanized mouse model of DMD [83]. Here, base editors were designed to skip a knocked-in pathogenic human exon 50, restoring the ORF and expression of a truncated but functional dystrophin.

#### 3.3.2. Prime Editors

Novel approaches that expand CRISPR/Cas editing platforms are driving advances in therapeutic precision editing for DMD. Prime editors extend the capabilities of base editors by coupling a catalytically impaired Cas9 endonuclease (nickase) to an engineered reverse transcriptase and a prime editing guide RNA (pegRNA), thereby enabling small insertions, deletions, and substitutions at targeted genomic sites without inducing DSBs or requiring a donor DNA template [84,85]. This strategy aims to increase fidelity of targeted corrections and reduce the stochastic indels common to CRISPR/Cas9-mediated NHEJ.

Chemello et al. were the first to report dystrophin rescue using a prime editing approach, demonstrating the correction of one of the most frequent *DMD* deletion mutations, exon 51 (ΔEx51), by inserting two nucleotides that reestablished the ORF in cardiomyocytes derived from human iPSCs [86]. In addition to restoring dystrophin expression, this editing approach normalized cardiomyocyte contractile abnormalities [87]. Subsequent studies have leveraged prime editing to correct specific point mutations, such as the c.428G>A point mutation in exon 6, achieving correction efficiencies of up to 28% [88]. Notably, more recent work has further advanced the capacity of prime editors to rescue dystrophin through improved delivery strategies. Using innovative viral gene-depleted adeno vector particles (AdVPs), investigators efficiently corrected human myogenic cells, underscoring the potential of this system as a flexible correction platform [89]. Despite these proof-of-concept data, achieving therapeutically relevant body-wide muscle editing remains an active and significant area of development.

Taken together, gene editing remains one of the most conceptually powerful therapeutic strategies in the DMD landscape because it offers the possibility of permanent correction at the endogenous locus. However, the translational gap remains substantial. Beyond the fundamental challenge of efficient whole-body delivery, concerns regarding long-term genomic stability, potential off-target activity, and the robust immunogenicity of bacterially derived Cas nucleases remain clinical constraints [90]. Furthermore, editing post-mitotic skeletal and cardiac muscle presents unique physiological hurdles regarding the durability of corrected nuclei during ongoing myofiber turnover. Thus, while genome editing may ultimately redefine the DMD therapeutic landscape, at present it remains a rapidly advancing yet largely preclinical or early translational strategy that requires further development of delivery vehicles and molecular precision.

### 3.4. Current Development Programs

The translational development of gene-editing tools is increasingly focused on optimizing nuclease activity, sequence specificity, and targeted tissue delivery. Nuclease engineering, guided by rational design and directed evolution, has produced high-fidelity Cas9 variants that nearly eliminate off-target editing, a key safety consideration for local and systemic administration, particularly critical in pediatric applications [76,91]. In parallel, delivery remains a central bottleneck: the large mass of skeletal muscle, the need for cardiac targeting, and the immunologic constraints associated with systemic viral vectors collectively complicate translation of these systems into effective therapeutics [68,92].

Most in vivo DMD editing programs still rely on AAV vectors to deliver the nuclease and guide RNA components due to their muscle tropism and established manufacturing pathways. However, systemic AAV delivery can require high vector doses for broad muscle transduction, raising concerns around immunogenicity, dose-dependent toxicity, and limited redosing [93,94]. Innate immune recognition of bacterial nucleases (including pre-existing adaptive immunity to commonly used Cas orthologs) further reinforces the need for transient expression strategies and careful selection of editing enzymes [95,96].

These constraints have motivated the exploration of alternative delivery systems, including lipid nanoparticles (LNPs), exosomes, and biosimilar extracellular vesicles (EVs). Industry efforts have placed particular emphasis on diversifying editing payload formats and expanding the capabilities of naturally occurring nuclease families [91,97]. For example, recently reported non-Cas9 editing nucleases, such as compact Cas12i (~1000 amino acids) and engineered enhanced optimized Cas12i (EOCas12i) enzymes, are being developed to improve the delivery of editing components and increase specificity in therapeutically relevant tissues. Notably, EOCas12i variants have demonstrated up to a 60-fold increase in editing efficiency over wild-type counterparts and enable the efficient multiplexed editing of up to 30 targets simultaneously [98,99,100]. Some of these novel systems have rapidly received patent approval and have advanced into clinical testing; for instance, the high-fidelity hfCas12Max nuclease is currently being evaluated in the first-in-human M.U.S.C.L.E. clinical trial (NCT06594094) for DMD [101]. This trial utilizes a single adeno-associated virus (AAV) vector to deliver HG302, which targets the exon 51 splice-donor site to induce exon skipping and restore functional dystrophin production [102]. Gene editing therapies discussed in this section are summarized in Table 3.

## 4. Emerging Modalities: Pharmacological and Biological Modulators

While genetic therapies have led recent clinical development in DMD, emerging pharmacological and biological strategies targeting downstream mechanisms represent promising mutation-agnostic interventions. These methods aim to lessen the effects of dystrophin deficiency by modulating inflammation, improving membrane stability, and reducing fibrosis, rather than directly restoring dystrophin. Unlike genetic tools, these approaches can be broadly used because membrane instability, chronic inflammation, and tissue remodeling are major drivers of DMD disease progression and are independent of specific gene mutations. Therefore, therapies targeting these pathways can be widely applied across the DMD patient population and may serve as foundational treatments that enhance the effectiveness of mutation-specific therapies. Key targets include inflammatory signaling pathways, especially those mediated by nuclear factor kappa B (NF-κB), which is a critical regulator of the chronic inflammatory response in dystrophin-deficient muscles. Emerging modalities discussed in this section are summarized in Table 3.

### 4.1. Pharmacological

#### 4.1.1. NF-κB Targeted Approaches

Chronic inflammation is a defining feature of DMD progression, and NF-κB signaling has been repeatedly implicated as a central transcriptional driver of inflammatory gene activation in dystrophin-deficient muscle from DMD mouse models and patients [103]. Consequently, modulation of the NF-κB pathway has been explored through both classical steroidal and emerging non-steroidal targeted approaches.

As discussed earlier, vamorolone represents a clinically validated treatment for DMD. It exemplifies an NF-κB–modulating anti-inflammatory with a distinct pharmacologic profile. Early preclinical work positioned vamorolone as a steroidal agent designed to retain anti-inflammatory efficacy while mitigating classical glucocorticoid side effects, including growth suppression and bone morbidity [104]. In clinical studies, vamorolone demonstrated efficacy and safety signals compared with the placebo and prednisone, supporting its development as a “dissociative” steroid alternative in DMD [44]. Longer follow-up and additional controlled datasets have continued to refine estimates of functional benefit and safety tradeoffs over extended treatment periods [45,105]. Consistent with this body of evidence, vamorolone received regulatory approval in 2023 for DMD, thereby formalizing its role as a next-generation anti-inflammatory standard of care for eligible patients [47].

In contrast, edasalonexent, a small-molecule NF-κB inhibitor, ultimately failed to demonstrate sufficient efficacy in late-stage clinical testing despite promising preclinical results in multiple animal models [106]. Although the mechanistic rationale for direct NF-κB inhibition remained strong, the PolarisDMD phase 3 trial (NCT03703882) did not meet its primary or secondary endpoints, limiting further clinical progression despite extensive development efforts [107]. This outcome highlights a recurring challenge in DMD drug development: where a targeted pathway rationale does not guarantee measurable functional benefit across such heterogeneous clinical cohorts, particularly over relatively short trial horizons [108].

#### 4.1.2. HDAC Inhibition: A Multi-Modal Anti-Inflammatory and Anti-Fibrotic Strategy

Beyond NF-κB, epigenetic modulation has also been explored to simultaneously influence inflammation, regeneration, and fibrosis. Histone deacetylase (HDAC) inhibition emerged as a compelling approach, following foundational preclinical studies demonstrating that HDAC inhibitors could activate myogenic programs and ameliorate dystrophic phenotypes in the *mdx* model [109]. Within this category, givinostat (DUVIZAT) is an orally bioavailable HDAC inhibitor developed specifically for DMD to provide multimodal benefits across inflammatory and fibrotic pathways while simultaneously promoting muscle regeneration [110]. Building upon this mechanistic rationale, givinostat received FDA approval in 2024, marking a major milestone for epigenetic modulation as a disease-modifying strategy in DMD [111,112]. Notably, this approval established the first non-steroidal, mutation-agnostic DMD treatment. As with other therapies that do not restore dystrophin expression, the key translational question remains how best to position HDAC inhibition. Is this approach a foundational background therapy, an adjunct to improve muscle quality for gene therapy recipients, or as a strategy to extend functional benefit in patients ineligible for current mutation-specific interventions.

From a translational standpoint, givinostat broadens the mutation-agnostic therapeutic landscape by targeting downstream pathology rather than attempting primary genetic rescue. While its regulatory approval is a landmark achievement, the ultimate clinical value of HDAC inhibition will be determined by its ability to meaningfully modify long-term functional trajectories when integrated into the evolving DMD standard of care. The challenge moving forward lies in quantifying the incremental benefit of HDAC inhibition in the context of current corticosteroid use and emerging genetic therapies.

### 4.2. Membrane Protectants

#### 4.2.1. Surfactant Copolymers (Poloxamers)

In addition to inflammatory and epigenetic modulation, there is growing interest in targeting the primary site of tissue fragility in DMD, the sarcolemma, which is the initiation site of several of the downstream pathological cascades. Since membrane instability precedes nearly all downstream pathophysiological events (e.g., calcium influx, necrosis, inflammation, and fibrosis), membrane-targeted therapies offer a mechanistically appealing, mutation-agnostic complement to existing genetic therapies.

One such membrane stabilization approach uses surfactant copolymers, most prominently poloxamer 188 (P188), which act as membrane sealants that can insert into and stabilize injured lipid bilayers of the sarcolemma [113]. This strategy is attractive in DMD because it does not require the delivery of genetic material and, in principle, can reduce acute membrane permeability, thereby mitigating calcium influx during mechanical stress.

In vitro studies have shown that multiple poloxamers, including P188, can enhance recovery from membrane injury in human non-muscle and mouse dystrophic muscle cells [114]. Moreover, preclinical in vivo studies have reported beneficial effects of P188 in dystrophin-deficient settings, including improved physiologic and histologic features in dystrophic muscle and diaphragm function in murine models under repeated dosing regimens [115]. Mechanistic and formulation studies also suggest that copolymer architecture and chemical end-group modifications can influence membrane interactions and stabilization behavior, potentially enabling the development of improved analogs with better tissue retention and activity [116]. Importantly, the P188 literature is not uniformly positive; some studies report limited efficacy or increased susceptibility to contraction-induced injury in dystrophic skeletal muscle [117]. This highlights that factors such as dosing, route, and tissue context can influence outcomes and should be considered in future development. Recent studies using novel bottlebrush block copolymers in the *mdx* mouse model provide additional support for the notion that these therapeutic approaches effectively mitigate disease progression. These studies report the in vivo rescue of skeletal muscle damage and the prevention of cardiac injury and death [118], underscoring that approaches aimed at reinforcing membrane resealing may require careful optimization, and that improvements in cardiac outcomes do not necessarily translate directly into skeletal muscle benefits across drug candidates [119].

Collectively, surfactant copolymers remain a biologically plausible but variably supported strategy, and further work is needed to define optimal dosing windows, target tissue distribution, and clinically meaningful endpoints that justify translation.

#### 4.2.2. TRIM72-Enhanced Membrane Repair

Alternative membrane-targeted approaches aim to reinforce the sarcolemma by enhancing the cell’s innate capacity to repair lesions after injury. One emerging approach leverages an endogenous protein essential for membrane repair in skeletal and cardiac muscle [120]. The tripartite motif protein 72/mitsugumin 53 (TRIM72/MG53) is a muscle-enriched TRIM protein that participates in acute membrane repair by recruiting intracellular vesicles and nucleating a repair patch at sites of sarcolemma injury [121]. TRIM72-based therapy is supported by studies demonstrating that recombinant human TRIM72 (rhTRIM72) protein supplementation enhances membrane repair capacity and reduces dystrophic pathology in vivo [122]. In the *mdx* mouse model, systemic administration of rhTRIM72 improved membrane integrity and reduced histopathologic indices of muscle damage, further supporting the idea that augmenting the repair response can partially compensate for dystrophin deficiency [123]. Broader preclinical studies across muscle injury and muscular dystrophy contexts further support the concept that TRIM72 supplementation can improve membrane stability and reduce myofiber injury burden, although the magnitude and durability of benefits appear to vary by model and dosing paradigm [124,125].

From a translational perspective, TRIM72-based interventions potentially offer several advantages over mutation-specific genetic correction approaches. First, enhancing the membrane repair response is mutation-agnostic, enabling eligibility across the entire DMD population [124]. Second, rhTRIM72 can be deployed as an adjunct therapy, potentially increasing the functional baseline upon which gene replacement strategies can be applied. This is particularly critical during early treatment windows when transgene expression and muscle remodeling are still evolving. Third, since membrane disruptions result from contraction-induced myofiber injuries, membrane protectants may offer more immediate mechanical benefits than approaches requiring long-term dystrophin replacement and tissue remodeling. Despite this preclinical rationale, human data on TRIM72-based therapeutics in DMD remain limited, with key translational questions regarding dose optimization, tissue penetration, and immune responses yet to be answered [125]. Recent advancements have introduced MyoTRIM, an engineered TRIM72-derived variant, as a next-generation membrane-repair biologic designed to overcome these hurdles [126]. Unpublished preclinical data indicate that MyoTRIM achieves therapeutic efficacy in DMD patient-derived cells and in a DMD mouse model. By enhancing protein stability and reducing off-target effects, MyoTRIM aims to provide a more durable therapeutic effect in dystrophic muscle, representing a significant evolution in the membrane-protection pipeline.

Collectively, enhancing membrane repair remains a biologically compelling strategy because it targets the primary mechanical defect in dystrophin-deficient muscle, a fragile sarcolemma. However, the current evidence base remains predominantly preclinical, and the extent to which improved membrane resealing translates into durable functional benefits in humans remains to be established. While emerging therapies like MyoTRIM and similar biologics offer a promising mutation-agnostic approach, their ultimate utility will be defined by their performance within a combinatorial regimen. By stabilizing the sarcolemma in an acute setting, these therapies may protect the muscle long enough for genetic therapies to achieve sustainable expression, potentially making them indispensable, albeit early-stage, components of the future DMD treatment landscape.

## 5. Clinical Outcome Measures and Interpretation in DMD Trials

The assessment of therapeutic efficacy in DMD clinical studies remains particularly challenging because disease progression is heterogeneous and trial durations are often short relative to the natural history of muscle decline [127]. while functional outcomes, such as the North Star Ambulatory Assessment (NSAA), the 6 min walk test (6MWT), and various timed function tests, remain the primary metrics for clinical significance, molecular biomarkers have gained increased weight in regulatory decision-making. Specifically, the quantification of dystrophin expression via Western blot or immunofluorescence is now frequently utilized as a surrogate endpoint to support accelerated approval pathways [128].

This reliance on molecular surrogates has created significant contention within the field. While dystrophin restoration is mechanistically aligned with disease correction, the relationship between restored protein levels and measurable functional benefit has not proven to be linear or consistently predictive in clinical settings [71]. This disconnect has intensified the debate surrounding regulatory approvals, particularly when therapies demonstrate robust biomarker signals but yield mixed or statistically insignificant functional endpoint data during pivotal reviews. Such discrepancies highlight the “biomarker-to-bedside” gap, in which a statistically significant increase in micro-dystrophin or exon-skipping-mediated protein rescue may not translate into improved ambulation or cardiopulmonary stability.

These challenges are especially pronounced when comparing mutation-specific modalities (exon skipping, micro-dystrophin gene replacement, and genome editing), each of which yields distinct expression profiles, tissue distributions, and durability of dystrophin rescue. Accordingly, future DMD trials will likely require more integrated, multi-domain frameworks that combine molecular persistence data with long-term respiratory, cardiac, and patient-reported outcomes. Only through longitudinal follow-up can the field better define the true therapeutic value of these high-cost interventions relative to the established natural history of the disease.

## 6. Discussion: Perspective Across Therapeutic Modalities and Future Outlook

As the therapeutic landscape for DMD continues to expand, it becomes increasingly important to not only establish the safety profiles of these interventions but also what each is realistically positioned to achieve. Mutation-specific approaches, such as exon skipping, micro-dystrophin gene replacement, and gene editing, aim to restore the missing functional dystrophin protein. However, they exhibit marked differences in clinical development and the evidence base for long-term benefit. Exon skipping is clinically validated but remains constrained by limited and variable dystrophin rescue (generally, <5% of healthy levels) and the logistical burden of lifelong intravenous dosing [56,57]. By comparison, AAV-mediated gene replacement offers broader application potential and more robust initial protein expression, yet faces significant regulatory and biological scrutiny regarding its long-term durability and the risk for severe, dose-limiting immune toxicities. While genome editing represents a conceptually permanent strategy, the absence of long-term human safety data and the inherent risk of off-target effects position it as the least clinically mature modality.

In contrast, mutation-agnostic approaches, including corticosteroids, vamorolone, givinostat, and emerging membrane-protective therapeutics, do not aim to restore dystrophin but instead target various cellular pathways to improve the biological environment of the affected muscles. Their broad applicability across genotypes positions them as attractive foundational therapies. It is increasingly evident that the future of DMD treatment likely resides in the synergistic use of these modalities. This emerging “combination paradigm” is founded on the rationale that partial dystrophin restoration (via gene therapy or exon skipping) may be most effective when coupled with agents that stabilize the sarcolemma or suppress chronic inflammatory signaling. This shift reflects a practical recognition that DMD exhibits multisystem pathophysiology, in which the meaningful stabilization of functional trajectories requires addressing both the primary genetic defects and their downstream degenerative consequences.

A critical enabling factor for next-generation genetic and biologic therapies for DMD will be improved delivery. While AAV vectors remain highly effective for muscle transduction, challenges such as pre-existing immunity and the “one-and-done” limitation of no-integrating episomes continue to drive the development of non-viral delivery platforms [129]. Lipid nanoparticles (LNPs) are particularly attractive for nucleic acid delivery and have demonstrated the feasibility of repeat-dose genome editing in vivo, offering a potential path toward more flexible therapeutic regimens [130,131]. Similarly, strategies utilizing engineered extracellular vesicles or exosomes may further expand tissue targeting while reducing systemic immunogenicity [132,133]. However, overcoming the muscle-specific biodistribution hurdle remains the primary translational barrier for these non-viral systems, as achieving body-wide efficient delivery to skeletal muscles remains a challenge [129,132].

## Figures and Tables

**Table 1:** Comparative overview of current and emerging therapeutic strategies for Duchenne muscular dystrophy (DMD): mutation requirement, durability, and key limitations.

Therapy Class	Mutation Requirement	Therapeutic Rationale	Durability	Key Limitations
Supportive Care	Mutation agnostic	Symptom management, preserves organ function with respiratory + cardiac support	Short term (maintenance dosing)	Does not address underlying disease
Corticosteroids	Mutation agnostic	Anti-inflammatory, delays muscle degeneration	Short term (maintenance dosing)	Significant side effects (growth, bone, metabolic, endocrine)
Dissociative Steroid	Mutation agnostic	NF-κB modulation, delays progression with reduced adverse side effects	Short term (maintenance dosing)	Adverse side effects reduced not eliminated Long-term benefit still being defined
Exon Skipping (ASO)	Mutation specific	Restores reading frame to produce truncated dystrophin	Transient (repeat dosing required)	Limited dystrophin restoration, repeated dosing, variable efficacy
Gene Replacement (AAV microdystrophin)	Mutation agnostic	Delivers microdystrophin gene via AAV to restore sarcolemma stability	Long-term proposed (single dose)	AAV immunogenicity, dose toxicity, limited re-dosing, waning transgene persistence
CRISPR/Cas Genome Editing	Mutation specific	Permanent genomic correction via exon deletion or reading frame restoration	Permanent proposed (single dose)	Off-target risk, AAV immunogenicity, delivery challenges
Non-viral Gene Editing Delivery	Mutation specific	Non-AVV delivery of editing machinery	Permanent proposed (possibly repeatable)	Low delivery efficiency, biodistribution challenges
NF-κB Inhibitors (non-steroidal)	Mutation agnostic	Reduces inflammation/fibrosis for improved muscle function	Short term (maintenance dosing)	Limited endpoint efficacy in trials
HDAC Inhibitor	Mutation agnostic	Epigenetic modulation reduces fibrosis/inflammation, activates satellite cell regeneration	Short term (maintenance dosing)	Modest functional benefit, long-term safety, off-target systemic effects
Membrane Stabilizers (Protein-based)	Mutation agnostic	Enhances membrane repair after injury, reduces pro-fibrosis/inflammation responses	Short term (maintenance dosing)	Limited human data, dosing schedule questions
Membrane Sealants (Polymers)	Mutation agnostic	Physical stabilization of sarcolemma	Short term (maintenance dosing)	Limited efficacy, transient benefit

**Table 2:** Clinical and standard-of-care therapeutic strategies for Duchenne muscular dystrophy (DMD): mechanism, mutation coverage, and regulatory status.

Therapy	Mechanism of Action	Mutation Coverage	Regulatory Status	Supporting Trial Phase (Clinical Trial Number)
**Supportive Care** **—** **Respiratory**				
Non-Invasive Ventilation (BiPaP)	Delivery of positive airway pressure ventilation to support respiratory function and reduce hypoventilation	Broad (mutation-agnostic)	Standard of Care	
Mechanical insufflation–exsufflation	Administration of positive pressure followed by rapid negative pressure to simulate cough and enhance airway clearance	Broad (mutation-agnostic)	Standard of Care	
Lung Volume Recruitment	Augments inspiratory capacity to improve lung compliance and prevent atelectasis	Broad (mutation-agnostic)	Standard of Care	
**Supportive Care** **—** **Cardiac**				
ACE Inhibitors	Inhibition of angiotensin-converting enzyme for reduced cardiac overload and pathological remodeling	Broad (mutation-agnostic)	Standard of Care	
Beta-Blockers	Blocks β-adrenergic receptors to reduce hear rate and myocardial workload	Broad (mutation-agnostic)	Standard of Care	
Cardiac Monitoring (Echocardiography, Cardiac MRI)	Surveillance of cardiac function for early detection of cardiomyopathy	Broad (mutation-agnostic)	Standard of Care	
**Supportive Care** **—** **Physiotherapy**				
Range-of-motion (ROM) Stretching	Regular stretching and ROM exercises to prevent muscle shortening and join contractures	Broad (mutation-agnostic)	Standard of Care	
Orthotic Support	Devices that maintain joint functional alignment with prolonged passive stretching	Broad (mutation-agnostic)	Standard of Care	
**Corticosteroids**				
Prednisone	Glucocorticoid receptor agonism with NF-κB inhibition to reduce inflammation Stabilizes sarcolemma and reduces muscle fiber degeneration	Broad (mutation-agnostic)	Standard of Care	
Deflazacort	Glucocorticoid receptor agonism with NF-κB inhibition to reduce inflammation Stabilizes sarcolemma and reduces muscle fiber degeneration	Broad (mutation-agnostic)	FDA Approved (2017) Expanded (2019)	Phase 3—(MP-104-NM-001) Observational—(NCT00468832)
**Dissociative Steroid**				
Vamorolone	Dissociative glucocorticoid receptor modulator with NF-κB inhibition Retains anti-inflammatory effects with reduced steroid-associated side effects Stabilizes sarcolemma for reduced muscle damage	Broad (mutation-agnostic)	FDA Approved (2023)	Phase 2b—VISION-DMD—(NCT03439670)

**Table 3:** Preclinical therapeutic development landscape for Duchenne muscular dystrophy (DMD): mechanism, mutation coverage, regulatory and clinical development status.

Therapy	Mechanism of Action	Mutation Coverage	Regulatory Status	Supporting Trial Phase (Clinical Trial Number)
**Antisense Oligonucleotide (ASO) Exon Skipping**				
Eteplirsen (Exondys 51)	Binds exon 51 for exon skipping to restore reading frame and produce truncated partially functional dystrophin	Mutation-specific (~13%)	FDA Accelerated Approval (2016)	Phase 2—Study 201—(NCT01396239) Extension—Study 202—(NCT01540409)
Golodirsen (Vyondys 53)	Binds exon 53 for exon skipping to restore reading frame and produce truncated partially functional dystrophin	Mutation-specific (~8%)	FDA Accelerated Approval (2019)	Phase 1/2—Study 4053-101—(NCT02310906)
Vitolarsen (Viltepso)	Binds exon 53 for exon skipping to restore reading frame and produce truncated partially functional dystrophin	Mutation-specific (~8%)	FDA Accelerated Approval (2020)	Phase 2—NCNP-01-201 (NCT02740972) Extension—NCNP-01 (NCT03167255)
Casimersen (Amondys 45)	Binds exon 45 for exon skipping to restore reading frame and produce truncated partially functional dystrophin	Mutation-specific (~8%)	FDA Accelerated Approval (2021)	Phase 3—ESSENCE—(NCT02500381)
**Gene Replacement (AAV microdystrophin)**				
Delandistrogene Moxeparvovec (Elevidys)—[Sarepta]	AAVrh74 delivery of micro-dystrophin transgene to produce truncated, semi-functional form of dystrophin	Broad (mutation-agnostic)	FDA Accelerated Approval (2023) Expanded (2024)	Phase 1/2—(NCT03769116) Phase 3—EMBARK—(NCT05096221)
Fordadistrogene Movaparvovec (PF-06939926)—[Pfizer]	AAV9 delivery of mini-dystrophin transgene to produce a truncated, semi-functional form of the dystrophin protein in muscle cells	Broad (mutation-agnostic)	Failed Phase 3—Discontinued	Phase 3—CIFFREO—(NCT04281485)
RGX-202—[Regenxbio]	AAV8 delivery of micro-dystrophin transgene to produce truncated semi-functional form of dystrophin with CT domain	Broad (mutation-agnostic)	In clinical development Fast Track Designation	Phase 1/2/3—AFFINITY DUCHENNE—(NCT05693142)
SGT-003—[Solid Biosciences]	AAV delivery of micro-dystrophin transgene to produce truncated semi-functional form of dystrophin that binds nNOS	Broad (mutation-agnostic)	In clinical development Fast Track Designation	Phase 1/2—INSPIRE DUCHENNE—(NCT06138639) Phase 3—IMPACT DUCHENNE—(NCT07160634)
GNT-0004—[Genethon]	AAV8 delivery of micro-dystrophin transgene to produce truncated semi-functional form of dystrophin	Broad (mutation-agnostic)	In clinical development PRIME Designation	Phase 1/2/3—GNT-016-MDYF—(2020-002093-27) Observational—(NCT03882827)
**CRISPR/Cas Genome Editing**				
Cas9	AAV delivery of CRISPR/Cas9 genome editing machinery to induce targeted DNA double-strand breaks and restore dystrophin reading frame via exon deletion or reframing	Mutation-specific (~60%)	Early clinical/preclinical	No active registered Cas9-only DMD clinical trials
Cas12i	AAV delivery of compact CRISPR/Cas12i nuclease enabling targeted genome editing to restore the dystrophin reading frame with improved packaging efficiency	Mutation-specific (~60%)	Early clinical/preclinical	No active registered Cas12i-only DMD clinical trials
hfCas12Max HG302—[HuidaGene]	AAV delivery of high-fidelity CRISPR/hfCas12Max nuclease engineered for enhanced specificity and multiplex genome editing to restore the dystrophin reading frame	Mutation-specific (~60%)	In clinical development Fast Track Designation	Phase 1—MUSCLE—(NCT06594094)
GEN6050X—[Peking Union Medical College Hospital]	Dual AAV9 delivery of base editing machinery to enable specific nucleotide conversion and restore the dystrophin reading frame without double-strand DNA breaks	Mutation-specific (~60%)	In clinical development	Phase 1—GATx-01-IIT-CLINC—(NCT06392724)
PBGENE-DMD—[PrecisionBio]	AAV9 delivery of ARCUS nuclease to excise DMD mutation hotspot region, restore the reading frame, and enable expression of a truncated but functional dystrophin	Mutation-specific (~60%)	In clinical development Fast Track Designation	Phase 1/2a—FUNCTION-DMD (NCT07429240)
**Non-viral Gene Editing Delivery**				
Lipid Nanoparticles (LNP)	Non-viral lipid-nanoparticle-mediated delivery of gene editing cargo to enable transient genome editing without AAV vectors	Mutation-specific (~60%)	Preclinical	No active registered DMD-specific LNP clinical trials
Extracellular Vesicles (EV)	Biological vesicle-mediated delivery of gene editing cargo to target tissues, enabling non-viral and potentially tissue-specific genome editing	Mutation-specific (~60%)	Preclinical	No active registered DMD-specific EV clinical trials
**NF-κB Inhibitors (non-steroidal)**				
Edasalonexent	Selective NF-κB inhibition to reduce inflammation and downstream muscle degeneration	Broad (mutation-agnostic)	Clinical development discontinued	Phase 3—PolarisDMD—(NCT03703882)
**HDAC Inhibitor**				
Givinostat (DUVYZAT)	HDAC inhibition to reduce inflammation and fibrosis and promote muscle regeneration	Broad (mutation-agnostic)	FDA Approved, (2024)	Phase 3—EPIDYS—(NCT02851797)
**Membrane Stabilizers (Protein-based)**				
TRIM72	Enhances sarcolemmal membrane repair via vesicle recruitment and repair patch formation	Broad (mutation-agnostic)	Preclinical/early translational development	No active registered TRIM72 DMD clinical trials
MyoTRIM	Engineered TRIM protein that enhances sarcolemmal repair and resealing capacity with reduced off-target metabolic risks	Broad (mutation-agnostic)	Preclinical/early translational development	No active registered MyoTRIM DMD clinical trials
**Membrane Sealants (Polymers)**				
P-188 NF (Poloxamer 188)—[Phrixus Pharmaceuticals]	Amphiphilic copolymer that inserts into damaged membranes to stabilize the sarcolemma and reduce membrane permeability	Broad (mutation-agnostic)	Clinical development discontinued	Phase 2—(NCT03558958)

## Data Availability

No new data were created or analyzed in this study. Data sharing is not applicable to this article.
